# Cutaneous IgA vasculitis triggered by acetaminophen

**DOI:** 10.1016/j.jdcr.2025.09.046

**Published:** 2025-10-27

**Authors:** Jose Gaspar de Alba, Aaron Cheng, Cadfael Soulard, Emily Ames, Victoria G. Farley

**Affiliations:** a Long School of Medicine, University of Texas Health San Antonio, San Antonio, Texas; b Kirk Kerkorian School of Medicine at UNLV, Las Vegas, Nevada; c Vivida Dermatology, Las Vegas, Nevada

**Keywords:** acetaminophen, adult-onset, conservative treatment, IgA vasculitis

## Introduction

Immunoglobulin A vasculitis (IgAV), formerly known as Henoch-Schoenlein purpura, is a systemic vasculitis caused by immune-complex deposition in small vessels, primarily affecting the joints, kidneys, skin, and gastrointestinal tract.^1^ Common symptoms include diffuse palpable purpura, nausea, vomiting, arthralgias, and nephritis, depending on the severity of disease.^1^ Histologically, IgAV is considered a subtype of leukocytoclastic vasculitis due to its characteristic neutrophil-predominant perivascular infiltrates and fibrinoid vessel wall destruction.^2^ While IgAV may be idiopathic, it frequently follows an upper respiratory tract infection.^1^ Other triggers include neoplasms, autoimmune diseases, vaccines, and medications such as antibiotics and tumor necrosis factor-α blockers.^1^ Rarely, IgAV is associated with over-the-counter analgesics.^3^ Herein, we report the second known case of acetaminophen-induced IgAV.

## Case report

**Fig 1:**
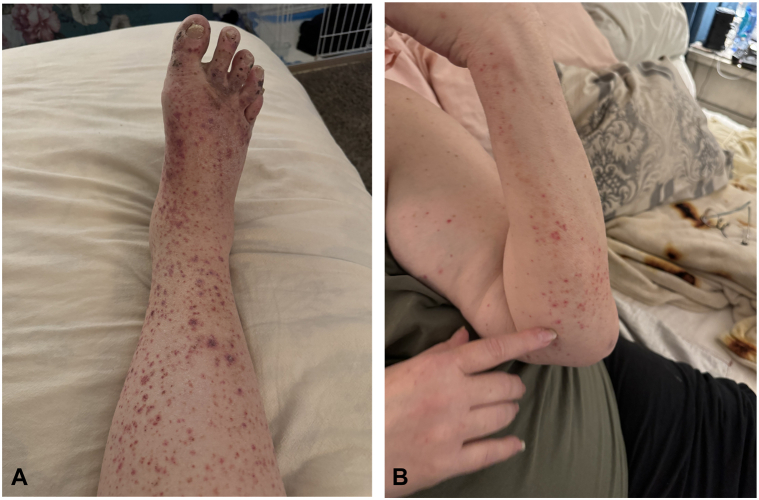
**A,** Clinical picture of palpable purpura with necrotic centers on the patients right lower extremity and **(B)**, patients left forearm.

A 61-year-old Caucasian female presented with pruritic and painful purpuric lesions on her right cheek, right zygoma, inferior forehead, right upper back, left forearm, and right pretibial region, which worsened with scratching (Fig 1, *A* and *B*). The patient has a history of type 2 diabetes mellitus and arthritic pain, which she managed with acetaminophen. She reported that the rash began 2 months prior and had been spreading, but it had started to clear since stopping acetaminophen.

Physical exam revealed palpable purpura with necrotic centers predominantly on the right lower extremity, suggestive of several different pathologies. Considerations for the differential diagnosis could include microscopic polyangiitis or a nonspecific small-vessel vasculitis, which may be drug-induced, infection-related, or idiopathic.

The patient had initially been referred to hematology by her primary care provider but had not yet had an appointment. Laboratory studies performed 3 weeks before presentation showed an elevated erythrocyte sedimentation rate (36 mm/h; reference: 0-15 mm/h), CRP (18 mg/dL; reference: <1.0 mg/dL), and leukocytosis (>11,000/mm^3^; reference: 4500-10,000/mm^3^). Notably, renal function tests were normal. Further autoimmune testing was unremarkable, with negative results for Jo-1 antibody, anti-SS-A, anti-SS-B, rheumatoid factor, antinuclear antibody, anti-dsDNA, Scl-70, RNP antibody, anti-Smith antibody, and cyclic citrullinated peptide antibody.

**Fig 2:**
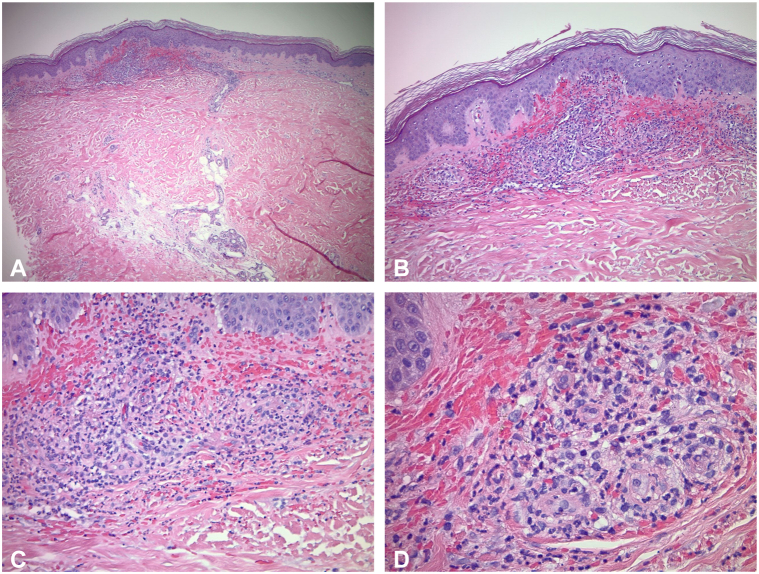
**A-D,** Histopathology depicting superficial perivascular lymphocytic infiltrates, neutrophils, and fibrinoid degeneration.

Two 4-mm punch biopsies were taken from the pretibial region, revealing superficial perivascular lymphocytic infiltrates, neutrophils, and fibrinoid degeneration, consistent with leukocytoclastic vasculitis (Fig. 2, *A*-*D*). Immunofluorescence confirmed granular IgA deposition in dermal blood vessels, diagnostic of IgAV.

The patient was instructed to avoid acetaminophen and prescribed triamcinolone acetonide 0.1% topical cream for itchiness. At her 10-day follow-up, she reported significant improvement despite not starting the prescribed therapy, noting that she had continued to avoid acetaminophen. Given her resolution, further testing was deemed unnecessary, though the patient was advised to follow up with her primary care provider for renal function testing and urinalysis over the next 6 months to ensure stability.

## Discussion

This case highlights the importance of recognizing IgAV as a rare but potential severe adverse reaction to acetaminophen. Nonspecific small-vessel vasculitis was considered, it can be idiopathic, but other causes include drugs, such as propylthiouracil or hydralazine, and infections, like HIV or Hepatitis C. Given the patient’s history and medication regimen, these causes were ruled out. Microscopic polyangiitis, an ANCA-associated vasculitis, was also considered due to its predilection for small vessels and potential renal involvement. However, the patient's renal function was normal, and she had no pulmonary symptoms such as alveolar hemorrhage, making microscopic polyangiitis less probable. For our case, ANCA titers were not obtained due to the absence of multisystem involvement. Our patient presented with a classic form of IgAV, with palpable purpura rather than the more severe and nonspecific lesions commonly seen in ANCA-associated vasculitis. For example, our case lacked severe ulcers and nodules often observed in ANCA-associated vasculitides. Additionally, our patient's clinical course was consistent with skin-limited disease that resolved after cessation of acetaminophen and did not involve systemic symptoms. Further diagnostic workup was therefore not pursued at the patient’s preference. Medically, it was agreed to observe her as she had no systemic symptoms. In contrast, ANCA-associated disease frequently demonstrates progressive renal, pulmonary, or neurological manifestations necessitating more aggressive diagnostic workup and potential immunosuppressive therapy, all of which were not indicated in our case.

A clear temporal association between acetaminophen use and IgAV was established in this case. The patient reported that the rash began 2 months prior while regularly using acetaminophen and had progressively worsened; after stopping acetaminophen without initiating any other treatment, the rash improved and eventually resolved completely with continued drug avoidance. This temporal association combined with histopathologic findings, supports drug-induced IgAV as the final diagnosis. Causality scores for the Adverse Drug Reaction Probability Scale by Naranjo and the WHO-Uppsala Monitoring Centre causality assessment system were Possible and Probable/Likely, respectively.^4^^,^^5^ Additionally, the severity level was assessed to be a level 2 on the Hartwig and Siegel Severity Assessment Scale.^6^

While topical corticosteroids are often used in treatment, this case emphasizes that discontinuing the offending drug alone can resolve symptoms. Acetaminophen-induced IgA vasculitis is rare, with only 1 other potential case reported in the literature. The previously reported case involved co-codamol (a combination of acetaminophen and codeine) and presented with a more severe clinical course, including significant renal involvement.^3^

IgAV primarily affects children, with an incidence of 3-26 per 100,000 individuals.^7^ In contrast, adult-onset IgAV is rarer, occurring in 1-5 per 100,000 individuals; in both cases there is a male predominance.^7^ The exact pathophysiology of IgAV remains unclear. Typically, IgA is produced at mucosal surfaces, but in IgAV, immune complexes containing IgA antibodies form in response to antigenic triggers, such as infections or medications.^8^ These complexes deposit in small vessels, leading to inflammation through the recruitment of mediators like prostaglandins. Additionally, the complement system is activated when C3-receptor lymphocytes bind to these immune complexes, further intensifying the inflammatory response. The mechanism of acetaminophen-induced IgAV may involve immune complex deposition and aberrant immune activation, with drug metabolites provoking an exaggerated immune response by mimicking pathogen-induce immunity.^1^

Supportive treatment is the primary intervention, as nearly 90% of adult-onset IgAV cases resolve spontaneously, as observed in our case.^9^ However, end-stage renal disease can develop in 5% to 15% of adult patients leading to worsened health outcomes.^7^ In cases of renal involvement, early aggressive therapy with high-dose corticosteroids and immunosuppressants is recommended.^9^^,^^10^

Clinicians should consider drug-induced IgAV when adult patients present with purpura, especially when systemic vasculitis features are absent. Early identification and prompt discontinuation of the drug can prevent unnecessary systemic immunosuppressant therapy and mitigate risks associated with misdiagnosis. Because of its widespread use, acetaminophen's role in adverse reactions may be underreported, with clinical symptoms commonly being linked to underlying infection or illness processes rather than the drug.

## Conflicts of interest

None disclosed.
